# Synthesis of ^18^O-labeled RNA for application to kinetic studies and imaging

**DOI:** 10.1093/nar/gkt344

**Published:** 2013-04-30

**Authors:** Tomohiro Hamasaki, Takahiro Matsumoto, Naoya Sakamoto, Akiko Shimahara, Shiori Kato, Ayumi Yoshitake, Ayumi Utsunomiya, Hisayoshi Yurimoto, Esteban C. Gabazza, Tadaaki Ohgi

**Affiliations:** ^1^Strategic Headquarters for Research and Development, BONAC Corporation, BIO Factory 4F, 4-1488 Aikawa, Kurume, Fukuoka 839-0861, Japan, ^2^Department of Immunology, Mie University Graduate School of Medicine, 2-174 Edobashi, Tsu, Mie 514-8507, Japan and ^3^Department of Natural History of Science, Hokkaido University Graduate School of Science, Kita-ku, Sapporo 060-0810, Japan

## Abstract

Radioisotopes and fluorescent compounds are frequently used for RNA labeling but are unsuitable for clinical studies of RNA drugs because of the risk from radiation exposure or the nonequivalence arising from covalently attached fluorophores. Here, we report a practical phosphoramidite solid-phase synthesis of ^18^O-labeled RNA that avoids these disadvantages, and we demonstrate its application to quantification and imaging. The synthesis involves the introduction of a nonbridging ^18^O atom into the phosphate group during the oxidation step of the synthetic cycle by using ^18^O water as the oxygen donor. The ^18^O label in the RNA was stable at pH 3–8.5, while the physicochemical and biological properties of labeled and unlabeled short interfering RNA were indistinguishable by circular dichroism, melting temperature and RNA-interference activity. The ^18^O/^16^O ratio as measured by isotope ratio mass spectrometry increased linearly with the concentration of ^18^O-labeled RNA, and this technique was used to determine the blood concentration of ^18^O-labeled RNA after administration to mice. ^18^O-labeled RNA transfected into human A549 cells was visualized by isotope microscopy. The RNA was observed in foci in the cytoplasm around the nucleus, presumably corresponding to endosomes. These methodologies may be useful for kinetic and cellular-localization studies of RNA in basic and pharmaceutical studies.

## INTRODUCTION

RNA-based drugs such as antisense oligomers, aptamers, short interfering RNA (siRNA) and microRNA (miRNA) have been under investigation for more than a decade. Since the key discoveries in RNA interference (RNAi) by Fire *et al.* (1), the pace of basic and clinical research on RNA drug candidates has been accelerating rapidly. To develop RNA as therapeutic drugs, effective analytical methods are needed for use in studies on the absorption, distribution, metabolism and excretion of RNA. However, new analytical methods for detecting labeled RNA drugs have not kept pace with research.

The conventional methods of labeling RNA are fluorescent and radioisotope labeling. While both of these methods have advantages, they also have disadvantages. Thus, fluorescent labeling is in principle sensitive enough to allow detection of a single molecule (2), but it has the disadvantage that the labeled RNA is not chemically equivalent to its unlabeled counterpart because of the covalently attached fluorophore. For example, blocking the 5′-hydroxyl terminus of the antisense strand of siRNA can lead to a loss of RNAi activity (3). Radioisotope labeling also has a high sensitivity and the label can be easily introduced into the molecule, for example, by transfer of the terminal phosphate of [5′-^32^P]ATP to the 5′-hydroxyl terminus of an RNA oligomer catalyzed by T4 polynucleotide kinase. The disadvantage of radioisotope labeling, however, is the risk associated with increased radiation exposure as well as the short half-life of some radioactive isotopes, in particular phosphorus.

With these considerations in mind, we set out to label RNA oligomers with the stable oxygen isotope ^18^O, which would avoid the disadvantages of fluorescent and radioisotope labeling outlined above. The properties of chemical equivalence and lack of radioactive decay make ^18^O well suited for labeling molecules for use as tracers in basic and clinical studies. Except for the labeling of proteins for quantitative proteomics (4,5) and the labeling of RNA for comparative mass-spectrometric analyses (6,7), ^18^O labeling has not been widely used in the life sciences. Several approaches to the labeling of nucleic acid oligomers with stable oxygen isotopes have been reported. These include the synthesis of 2′-^18^O-labeled uridine RNA (8) and the incorporation of ^18^O into the 3′-phosphate group of RNA on endonuclease digestion (6,7). In addition, ^18^O has been incorporated into the phosphate groups of nucleic acid dimers during liquid-phase synthesis by using ^18^O water as the oxygen donor to phosphate (9,10), and ^18^O has been incorporated into dTdT and ^17^O into one phosphate group of a deoxyribonucleotide octamer during solid-phase synthesis (11).

Here, we report the synthesis of 21mer RNA with ^18^O incorporated into all of the phosphate groups by using ^18^O water in the oxidation step during solid-phase synthesis. We demonstrate the stability of the ^18^O label under physiological conditions and the equivalence of ^18^O-labeled and unlabeled RNA. We then go on to show that isotope ratio mass spectrometry (IRMS) and isotope microscopy can be used for the quantification and imaging of ^18^O-labeled RNA in biological material.

## MATERIALS AND METHODS

### RNA synthesis

RNA oligomers were synthesized on commercially available controlled-pore glass solid supports with a pore size of 1000 Å (3-Prime, Aston, PA, USA) in columns installed in an Expedite model 8909 nucleic acid synthesizer (Applied Biosystems, Foster City, CA, USA). Synthesis was carried out on a 1-μmol scale from the *tert*-butyldimethylsilyl (TBDMS) amidites (ST Pharm, Seoul, Korea) as described by Bellon (12). The detritylation reagent was 3% trichloroacetic acid in dichloromethane. For the coupling reaction, TBDMS amidites were used as 0.05 M solutions in acetonitrile, and the activator was 0.25 M 5-benzylmercaptotetrazole in acetonitrile. The capping reagent was a mixture of 10% acetic anhydride in tetrahydrofuran (THF) and 10% *N*-methylimidazole in THF/pyridine (8:1), and the oxidizing agent was 0.01 M iodine in THF/pyridine/water (78:20:2). All reagents and solvents used were commercially available. ^18^O-labeled RNA oligomers were synthesized in the same way except that the solvent for the oxidizing agent was THF/pyridine/^18^O water (78:20:2). ^18^O-enriched water (99.2 atom %) was purchased from Taiyo Nippon Sanso (Tokyo, Japan). After synthesis, the protecting groups of the RNA oligomers were removed. The purity of the crude RNA product was confirmed by reverse-phase high-performance liquid chromatography (HPLC) and liquid chromatography coupled with electrospray ionization quadrupole time-of-flight tandem mass spectrometry (LC-ESI-Q-Tof MS). After ethanol precipitation, RNA oligomers were purified by preparative reverse-phase HPLC. Purified RNA oligomers to be used for the preparation of siRNA were dissolved in 30 mM HEPES-KOH buffer, pH 7.4, containing 100 mM potassium acetate and 2 mM magnesium acetate. siRNAs were prepared by annealing the purified short complementary strands by incubation at 90°C for 10 min and then at room temperature for 1 h. Duplex formation was confirmed by 15% polyacrylamide gel electrophoresis followed by staining with ethidium bromide.

### HPLC and LC-ESI-Q-Tof MS

HPLC was performed on a Shimadzu LC-10A system (Shimadzu, Kyoto, Japan) equipped with an XBridge Oligonucleotide Separation Technology (OST) C18 column (1.7 μm; 4.6 × 50 mm; Waters, Milford, MA, USA). The following solvent system was used for analytical and preparative HPLC: buffer A (50 mM triethylammonium acetate, pH 7, in 5% acetonitrile) and buffer B (50 mM triethylammonium acetate, pH 7, in 90% acetonitrile). Elution was carried out with a 5–10% linear gradient of buffer B in buffer A. Mass spectra were obtained with a Waters Acquity ultra performance liquid chromatography (UPLC) system coupled with a Waters Synapt G2 ESI-Q-Tof mass spectrometer. The UPLC system was used with a Waters Acquity UPLC OST C18 column (1.7 μm; 2.1 × 50 mm). The following solvent system was used for liquid chromatography–mass spectrometry: solvent A (an aqueous solution of 15 mM triethylamine and 400 mM hexafluoroisopropanol, pH 7.9) and solvent B (50% methanol). Elution was carried out with a 5–50% linear gradient of solvent B in solvent A. The mass spectrometer was operated in negative ion mode. External calibration was performed with sodium iodide, and the Transform software (Waters) was used for spectral deconvolution.

### Stability of ^18^O label at various pH values

Solutions of ^18^O-labeled uridine 20mer (20 μM) were prepared in 50 mM sodium phosphate buffer at pH 3, 4, 5.5, 7 and 8.5. Solutions were incubated at 37°C and samples were analyzed by LC-ESI-Q-Tof MS after 1, 3, 7, 14, 21 and 28 days.

### Circular dichroism spectra

Circular dichroism (CD) spectra of 10 μM siRNA solutions in 50 mM sodium phosphate buffer, pH 7.5, were recorded at 25°C with a JASCO J-720 W spectropolarimeter (JASCO, Tokyo, Japan) in a 1-cm-path-length quartz cell at a rate of 50 nm/min and digitized at a resolution of 0.1 nm. The bandwidth was 1 nm and the response time was 8 s. For each determination, at least five spectra were accumulated and the averaged spectrum was subjected to noise reduction.

### Determination of melting temperature (*T*_m_)

Melting curves of siRNA in 50 mM sodium phosphate buffer, pH 7.5, were recorded with a UV-1800 UV-VIS spectrophotometer (Shimadzu Scientific Instruments, Kyoto, Japan). The absorbance at 260 nm was monitored as the temperature was raised from 25 to 95°C at a rate of 0.5°C/min. *T*_m_ values were calculated with Tm Analysis Software (Shimadzu Scientific Instruments).

### Cell culture

The cell lines A549 (a human lung adenocarcinoma epithelial cell line) and HCT116 (a human colorectal carcinoma cell line) were obtained from the European Collection of Cell Cultures (Salisbury, UK). The cells were cultured in Dulbecco’s modified Eagle’s medium (DMEM) and McCoy’s 5A medium (Sigma, St. Louis, MO, USA), respectively, containing 10% heat-inactivated fetal bovine serum (MP Biomedicals Japan, Tokyo, Japan) and incubated at 37°C in a humidified 5% CO_2_ atmosphere.

### Quantitative real-time polymerase chain reaction

HCT116 cells (4 × 10^4^ cells) suspended in 0.4 ml DMEM containing 10% fetal bovine serum were transfected with 98.5 μl siRNA targeting the human *GAPDH* gene in Opti-MEM reduced-serum medium (Invitrogen, Carlsbad, CA, USA; final concentrations of 0.1, 0.3 and 1 nM) by using 1.5 μl Lipofectamine 2000 (Invitrogen) and then seeded on 24-well plates. Negative-control cells were treated with 100 μl Opti-MEM or 1.5 μl Lipofectamine 2000 plus 98.5 μl Opti-MEM (mock). Cells transfected with siRNA were examined 48 h after transfection and used for RNA analysis. Total RNA was extracted from the cultured cells with the RNeasy Mini Kit (Qiagen, Germantown, MD, USA), and cDNA was synthesized by using SuperScript III (Invitrogen) according to the supplier’s instructions. To determine the mRNA knockdown level, quantitative real-time polymerase chain reaction (PCR) was carried out with the following primers (Greiner Japan, Tokyo, Japan): human *GAPDH*, forward 5′-GGAGAAGGCTGGGGCTCATTTGC-3′ and reverse 5′-TGGCCAGGGGTGCTAAGCAGTTG-3′; and the human β-actin gene, forward 5′-GCCACGGCTGCTTCCAGCTCCTC-3′ and reverse 5′-AGGTCTTTGCGGATGTCCACGTCAC-3′. Synthesized cDNAs were mixed with primers in LightCycler Fast Start DNA Master SYBR Green I reaction mix (Roche Diagnostics, Tokyo, Japan). Quantitative real-time PCR was performed with a LightCycler DX400 instrument (Roche Diagnostics), and the reaction conditions were as follows: 40 cycles of denaturation at 95°C for 5 s, annealing at 62°C for 15 s and polymerase extension at 72°C for 15 s (human *GAPDH*), or 40 cycles of denaturation at 95°C for 5 s, annealing at 65°C for 15 s and polymerase extension at 72°C for 15 s (the human β-actin gene). The data obtained from the assays were analyzed with LightCycler Software version 4.1 (Roche Diagnostics). The mRNA knockdown levels of each sample were normalized to the β-actin transcript level. The experiments were performed in triplicate.

### Measurement of ^18^O/^16^O ratio of RNA by IRMS

^18^O/^16^O ratios were measured with a Delta V isotope ratio mass spectrometer (Thermo Fisher Scientific, Waltham, MA, USA) interfaced with a thermal conversion elemental analyzer (Thermo Fisher Scientific). Wild-type C57BL/6 male mice purchased from Nihon SLC (Hamamatsu, Japan) were maintained in Mie University’s animal house. The experimental protocol was approved by the Committee for Animal Investigation of Mie University. ^18^O-labeled control siRNA (100 or 500 μg) in water for injection was administered intravenously. Animals were sacrificed 5, 30 and 60 min after RNA administration, blood was collected in heparinized tubes and plasma was prepared by centrifugation at 900*g*. For the calibration curve, pooled plasma prepared from untreated mice was spiked with ^18^O-labeled control siRNA (final RNA concentrations: 0, 10, 20, 40, 60, 80,100 and 200 μg/ml). For IRMS, plasma samples were lyophilized. Experiments were performed in triplicate. The relative difference in ^18^O/^16^O ratio between the sample and the internal standard, δ^18^O, was expressed as parts per thousand according to the equation δ^18^O (‰) = [(*R*_sample _− *R*_standard_)/*R*_standard_] × 1000, where *R* denotes the ^18^O/^16^O ratio. The internal standard was Vienna Standard Mean Ocean Water.

### Imaging of cells transfected with ^18^O-labeled RNA by isotope microscopy

A549 cells were seeded on a piece of a silicon wafer (7 × 7 mm) in a 35-mm dish (5 × 10^5^ cells/dish) and incubated at 37°C in humidified 5% CO_2_. After 24 h, the culture medium was replaced by 2 ml of fresh medium and the cells were transfected with unlabeled or ^18^O-labeled control siRNA (490 μl) in Opti-MEM (final concentrations, 100 and 300 nM) by using Lipofectamine 2000 (10 μl). Twenty-four hours after transfection, the cells on the silicon wafer were washed with phosphate-buffered saline, immobilized with 2.5% glutaraldehyde in phosphate-buffered saline at 37°C for 1 h and dehydrated by soaking successively in 20, 40, 60, 70, 80, 90 and 100% ethanol followed by *tert*-butanol at room temperature for 10 min each. The transfected cells were frozen at 4°C for 24 h and dried under reduced pressure for isotope microscopy. Samples were coated with a 30-nm layer of gold to avoid the accumulation of positive charge due to the primary beam of the isotope microscope.

There are two approaches to obtaining an isotope map by secondary ion mass spectrometry (SIMS): scanning and direct imaging. In scanning, isotopic data are collected sequentially from each point of the analyzed area with a focused primary ion beam and images are reconstructed by computer. In direct imaging, stigmatic ion optics are used with a high-efficiency 2D stacked complementary metal–oxide–semiconductor (CMOS) active pixel sensor (SCAPS) detector. The SCAPS detector features a wide dynamic range, no dead time and direct detection of ions. Small structures in biological materials, such as organelles or vesicles, are not always exposed at the sample surface. Direct imaging is useful to reach targets on the order of a few tens of nanometers in size embedded in a matrix on the order of a few microns in size because a high-intensity primary beam can be used without degrading the spatial resolution.

In the present study, the Hokudai isotope microscope system (a Cameca ims-1270 ion microprobe equipped with a SCAPS detector; Cameca, Gennevilliers, France) was used to visualize the isotope distribution in the cells, a technique known as isotopography. The sample surface (∼100 × 100 µm^2^) was homogeneously irradiated with a 20-keV Cs^+^ primary beam with a beam current of ∼5 nA. Secondary ion images of ^12^C^14^N, ^16^O, ^18^O and ^16^O were obtained sequentially for the same field for each analytical sequence. The exposure times were 5 s for ^12^C^14^N, 5 s for ^16^O and 500 s for ^18^O isotopography. A 50-µm contrast aperture was used, and the secondary ion contributions, except for objective isotopes, were cut by the exit slit. The typical spatial resolution under the conditions of isotopography was ∼0.3 µm, and the width of a pixel of SCAPS corresponds to 0.2 µm on the sample surface. An image-processing method with a moving average of 3 × 3 pixels was applied to ^18^O/^16^O isotopography. The resulting spatial resolution of the isotope ratio image was 0.6 µm. Other analytical methods for isotopography were carried out as described elsewhere (13,14).

## RESULTS AND DISCUSION

### Synthesis of ^18^O-labeled RNA

**Scheme 1.**
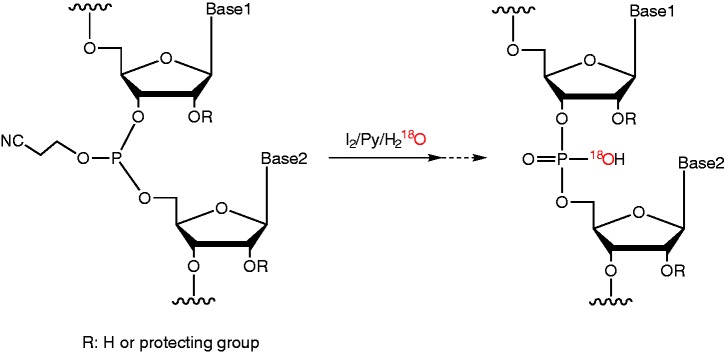
^18^O-labeling of oligonucleotides during the oxidation step.

**Figure 1.**
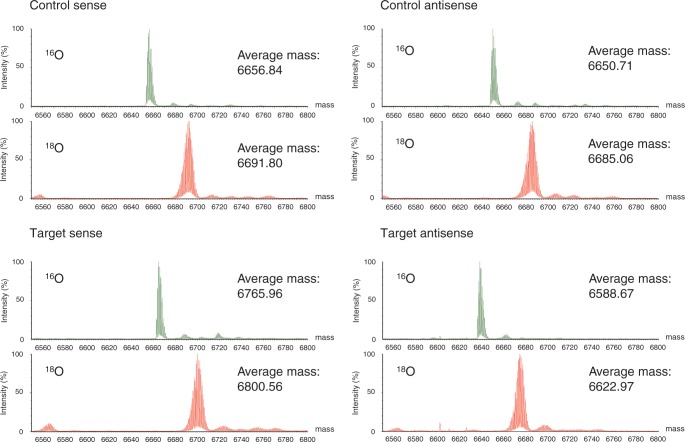
LC-ESI-Q-Tof mass spectra of control and target siRNA. Green spectra indicate ^16^O-RNA, and red spectra indicate ^18^O-labeled RNA.

We designed a siRNA targeting the human *GAPDH* gene (‘target siRNA’) and a control siRNA that had no gene-silencing target in the human genome, both 21 nucleotides in length (Table 1). The sense and antisense strands of each siRNA were separately synthesized in the solid phase by the phosphoramidite method with ^16^O or ^18^O water as the oxygen donor to phosphate at the oxidation step of the synthetic cycle (see Scheme 1). It was expected that the use of ^18^O water as the oxygen donor would yield RNA oligomer with one nonbridging ^18^O atom per phosphate group. On HPLC, the elution times of the ^16^O- and the ^18^O-RNA oligomers were indistinguishable (Supplementary Figure S1), and their yield and purity estimated by their absorbance at 260 nm were nearly identical (Table 1). Thus, the use of ^18^O water in the oxidation step achieved the same result as when ^16^O water was used. LC-ESI-Q-Tof mass spectra of the RNA oligomers (Figure 1) show that the mass difference between the ^18^O-labeled and the unlabeled oligomer was 34–35 Da (Table 1). This is somewhat lower than the mass difference calculated on the basis of the incorporation of 20 atoms of ^18^O into each 21mer allowing for the 99.2% isotopic purity of the ^18^O water used (2 × 20 × 0.992 = 39.68). This somewhat low value may be explained by a combination of an ^18^O/^16^O kinetic isotope effect and reaction with water from moisture in the air. The discrepancy may be decreased by using ^18^O water with a higher degree of isotopic purity and excluding moisture from the air. In any case, these results confirm that the ^18^O-labeled RNAs were correctly synthesized and that one ^18^O atom was incorporated into each phosphate group ∼85% of the time under our experimental conditions.

**Table 1.** Summary of RNA synthesis, HPLC analysis and mass spectrometric analysis

siRNA	Sequence (5′→3′)	Oxygen isotope	Yield (nmol)^a^	HPLC purity (%)^a^	Theoretical mass (Da)	Observed mass (Da)	Observed mass difference (Da)^b^
Control sense	UACUAUUCGACACGCGAAGUU	^16^O	473	89.1	6657.0	6656.8	34.96
		^18^O	484	87.5	6691.1	6691.8	
Control antisense	CUUCGCGUGUCGAAUAGUAUU	^16^O	425	75.3	6650.9	6650.7	34.35
		^18^O	443	74.5	6685.0	6685.1	
Target sense	CCAUGAGAAGUAUGACAACAG	^16^O	472	80.3	6766.1	6766.0	34.60
		^18^O	475	78.1	6800.2	6800.6	
Target antisense	GUUGUCAUACUUCUCAUGGUU	^16^O	469	79.1	6588.9	6588.7	34.30
		^18^O	475	84.4	6622.9	6623.0	

^a^Estimated by the absorbance at 260 nm.

^b^The difference between the observed mass of the ^18^O-labeled RNA and the observed mass of the unlabeled RNA. The theoretical mass difference is ∼39.68.

Limbach’s group (6,7) has described the incorporation of ^18^O into the 3′-phosphate groups of RNA during endonuclease digestion. However, this method can only be used on a laboratory scale, and the ^18^O label is incorporated only into the 3′-phosphate group. In addition, Dai *et al.* (8) have described the solid-phase synthesis of 2′-^18^O-labeled uridine RNA by using 2′-^18^O uridine phosphoramidite derived from 2,2′-cyclouridine. However, the required phosphoramidite is not commercially available, and labeling is limited to uridine residues. The incorporation of ^18^O into the phosphate group of nucleic acid oligomers by using ^18^O water as an oxygen donor to phosphate has been reported by Potter’s group in the liquid-phase synthesis of the dimers dGdA (9) and rUrA (10) for their studies on oxygen-chiral phosphate groups. Although ^18^O has been incorporated into dTdT and ^17^O into one phosphate group of a deoxyribonucleotide octamer by the phosphoramidite solid-phase method (11), our study is, to the best of our knowledge, the first in which a stable oxygen isotope has been incorporated into multiple phosphate groups in a nucleic acid oligomer.

By the method reported here, ^18^O can be incorporated into all phosphate groups in an RNA oligomer or, by limiting the use of ^18^O water to certain oxidation steps during synthesis, only into selected phosphate groups. The method could also be used to incorporate ^18^O into DNA oligomers. Similarly, the method could be extended to the use of ^34^S-labeled sulfurizing reagents such as 3*H*-1,2-benzodithiol-3-one 1,1-dioxide (Beaucage reagent) (15,16) or ^10^B-labeled *N*,*N*-diisopropylethylamine/borane complex (17) in the oxidation step to allow the synthesis of ^34^S-labeled phosphorothioate or ^10^B-labeled boranophosphate oligomers. Thus, this labeling approach is not limited by the nature of the phosphoramidites, labeling position, nucleotide sequence or class of oligomer, and it should allow the practical preparation of labeled oligonucleotides with any solid-phase DNA/RNA synthesizer. The approach is readily amenable to scaling up, and the handling of the stable isotopes is simple and safe.

### Stability of the ^18^O label in the RNA oligomer

The stability of the ^18^O label in the RNA oligomer under physiological conditions is an important consideration. Any back exchange between ^18^O-labeled RNA and the surrounding ^16^O water would result in less accurate quantitative analysis and less sensitive imaging, thus limiting the value of the labeling technique. In the absence of ribonuclease, ^18^O/^16^O exchange is not observed in 3′-labeled RNA up to 8 h (18). To test the stability of our ^18^O label under physiological conditions over longer periods, ^18^O-labeled uridine 20mer was synthesized, dissolved in buffers at pH values of 3–8.5, and incubated at 37°C for various times. Because RNA is known to be unstable under alkaline conditions, pH values >8.5 were not tested. Mass spectra of the samples after incubation for 28 days are shown in Supplementary Figure S2. At all pH values, the ^18^O-labeled 20mer showed the same isotope-distribution pattern and average molecular weights of 6093.7–6094.0, which are indistinguishable from the theoretical average of 6094.0. Thus, no significant ^18^O/^16^O exchange occurred in the pH range 3–8.5 at 37°C over periods of up to 28 days. The ^18^O label in RNA oligomers is therefore expected to be stable under physiological conditions, so that from this point of view ^18^O-labeled RNA oligomers should be suitable for use as tracers in clinical studies.

### Equivalence of ^18^O-labeled and unlabeled RNA

For labeled RNA to be used as a tracer, it should ideally have the same physicochemical and biological properties as the corresponding unlabeled RNA. To demonstrate the equivalence of ^18^O-labeled and unlabeled RNA, we prepared target and control siRNAs in which labeled and unlabeled sense and antisense strands were paired in all four combinations: ^16^O sense with ^16^O antisense (^16^O/^16^O), ^16^O sense with ^18^O antisense (^16^O/^18^O), ^18^O sense with ^16^O antisense (^18^O/^16^O) and ^18^O sense with ^18^O antisense (^18^O/^18^O). On polyacrylamide gel electrophoresis, target and control siRNAs with all combinations of labeled and unlabeled strands showed a single band corresponding to a double-stranded oligomer ∼20 bp in length (Supplementary Figure S3). This shows that the ^18^O-labeled RNA oligomers annealed normally to both ^18^O-labeled and unlabeled complementary strands to form a duplex.

Melting curves were acquired for the four siRNA duplex combinations (data not shown) and used to calculate *T*_m_ values (Supplementary Table S1). All of the target siRNA duplex combinations had the same *T*_m_ value to within 0.6°C and all of the control siRNA duplex combinations to within 0.5°C. Thus, ^18^O labeling of the backbone phosphate groups had little or no effect on the strength of base pairing.

Furthermore, the CD spectra of the four target and control siRNA duplex combinations were all similar, with a negative maximum at 210 nm and a positive maximum at 260 nm (Supplementary Figure S4). This CD spectral pattern typically indicates the A-form RNA duplex (19,20). Thus, the CD spectra show that the ^18^O-labeled siRNA strands paired with either labeled or unlabeled strands to form an A-type helix that was conformationally indistinguishable from that formed by two unlabeled strands.

**Figure 2.**
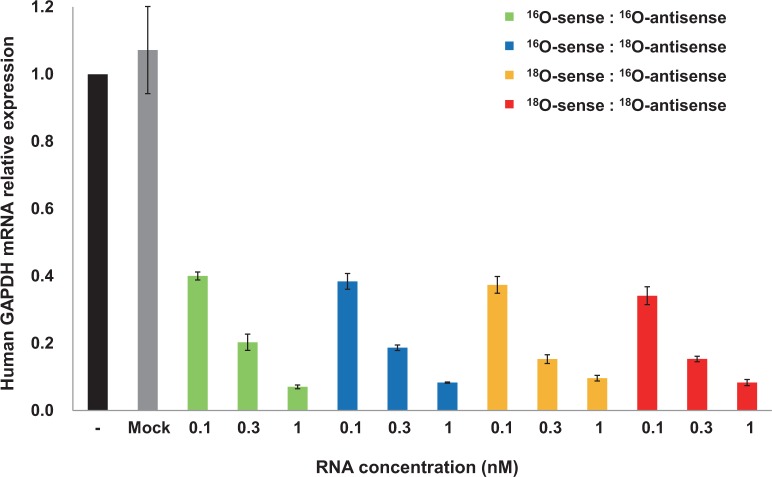
RNAi activity of target siRNA. Each siRNA was transfected into A549 cells at the indicated concentrations by using Lipofectamine, and human GAPDH mRNA levels were determined by quantitative real-time PCR. Each value is the average of three independent experiments, and the error bars indicate the standard error.

Finally, to compare the biological activity of ^18^O-labeled and unlabeled siRNA, we measured the silencing effect of siRNAs targeting the *GAPDH* gene in the human cell line HCT116. These short double-stranded RNAs suppress the expression of their target genes by causing cleavage of the mRNA or inhibiting its translation (21–24). As expected, the control siRNA had no silencing effect (data not shown). In contrast, the target siRNA had a silencing effect, and the effect of the ^18^O/^18^O target siRNA was dose-dependent and just as strong as that of the ^16^O/^16^O target siRNA (Figure 2). In RNAi, the antisense strand is the main effective strand (25,26), and our ^16^O/^18^O siRNA, which had the ^18^O label only in the antisense strand, also had a silencing effect that was indistinguishable from that of ^16^O/^16^O-siRNA.

Taken together, these results show that the physicochemical properties and biological activity of the ^18^O-labeled RNA were essentially the same as those of the unlabeled RNA, increasing our confidence in the suitability of ^18^O-labeled RNA as a tracer.

### Quantification of ^18^O-labeled RNA

**Figure 3.**
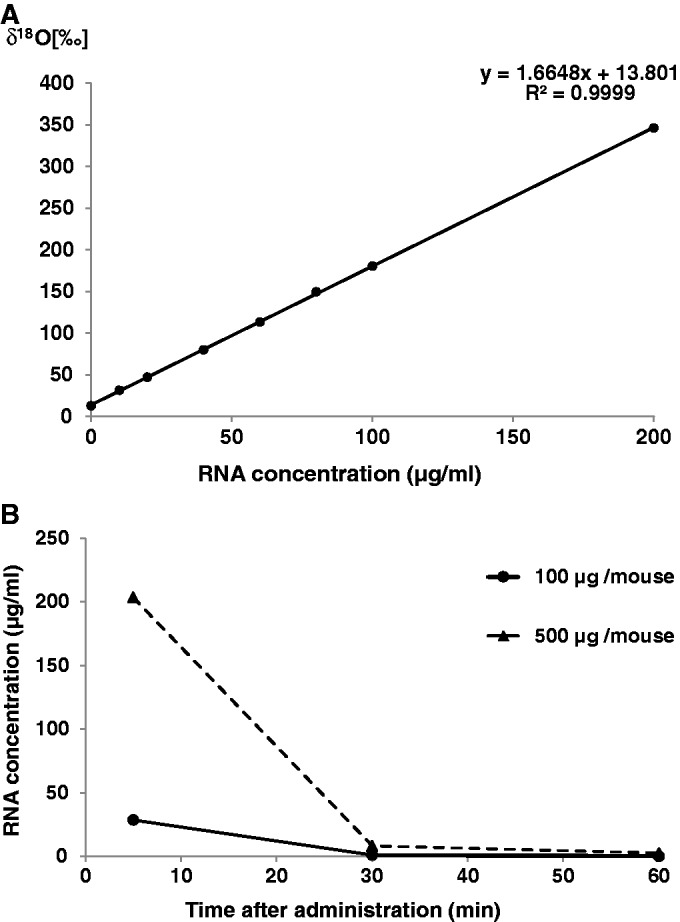
Determination of siRNA in the blood of mice after intravenous administration. (**A**) ^18^O/^16^O isotope ratio as a function of ^18^O-labeled control siRNA concentration. δ^18^O values were calculated from the results of IRMS, and the concentration of ^18^O-labeled control siRNA in the plasma was determined by its absorbance at 260 nm. Each value is the average of at least three independent experiments. (**B**) Blood concentration-time curve of control siRNA. The RNA concentrations were calculated from the δ^18^O values of the plasma samples. Each value is the average of at least three independent experiments.

IRMS, which can be used to determine the ^18^O/^16^O ratio in samples, finds application in food science (27) and sports drug testing (28). In the present study, we extended this technique to estimate the concentration of ^18^O-labeled siRNA in biological samples. A calibration curve for ^18^O-labeled control siRNA in mouse plasma was constructed in which δ^18^O, the relative difference in ^18^O/^16^O ratio between the sample and the internal standard, was estimated by IRMS and plotted against the concentration of control siRNA as determined by its absorbance at 260 nm (Figure 3A). The calibration curve showed good linearity over the entire concentration range of 0–200 μg/ml, with a correlation factor (R^2^) of 0.9999. We next estimated the blood concentration of control siRNA after intravenous administration to mice. Naked RNA oligomer in the blood is rapidly degraded by ribonucleases and eliminated by the kidney. After administration of naked RNA oligomer (100 or 500 μg), the blood concentration-time curves (Figure 3B) showed a rapid decay of the signal that presumably reflects the degradation of the RNA. These results give us confidence that ^18^O labeling combined with IRMS allows determination of the blood concentration of RNA after administration, opening the way for the use of ^18^O labeling in pharmacokinetic studies of RNA drugs.

To detect intact RNA after administration, it is necessary to protect it from degradation, for example, by chemical modification or a drug delivery system. In this study, the administered RNA was not protected, so it is assumed that the observed ^18^O signal came not only from the intact RNA but also from its degradation products. However, even with chemical modification or a drug-delivery system, it is in general difficult to ensure the complete stability of RNA oligomers *in vivo*. In addition, it is difficult to judge the intactness of an RNA oligomer *in vivo* by any labeling method.

Isotope dilution mass spectrometry has previously been used for the quantification of a therapeutic monoclonal antibody containing ^13^C and ^15^N (29) and an antiretroviral drug containing ^13^C (30), with the isotopically labeled compound as the internal standard in both studies. However, it is difficult to apply such isotopic-labeling methods to the analysis of large molecules such as proteins and oligonucleotides because the mass difference between the unlabeled compound and the isotopically labeled compound cannot be reliably determined without additional treatment such as enzymatic digestion or fragmentation (6,29). Immunoassay and radioisotope labeling are sometimes used for the quantification of oligonucleotides, but the former method requires the preparation of a specific antibody for each compound, while the latter carries the risks associated with increased radiation exposure. Although our method of detection of ^18^O-labeled RNA may still need improvement in sensitivity and accuracy, the ^18^O-labeling approach described here is likely to be useful in basic and clinical studies of RNA drugs.

### Imaging of ^18^O-labeled RNA

Isotope microscopy is based on SIMS coupled with a special ion imager and is capable of 2D ^18^O/^16^O isotopic analysis (13,14). SIMS is used to analyze the composition of solid surfaces and thin films by irradiating the surface of the specimen with a focused primary ion beam and collecting and analyzing the ejected secondary ions. The secondary ions can be measured with a mass spectrometer to determine the elemental, isotopic or molecular composition of the surface. Isotope microscopy has been used mainly in cosmochemistry and geochemistry for the isotopic elemental analysis of extraterrestrial minerals such as primitive carbonaceous chondrites (31), lunar rocks (32) and material returned from the asteroid Itokawa by the Hayabusa mission (33). These techniques have yet to find application in the pharmaceutical field.

**Figure 4.**
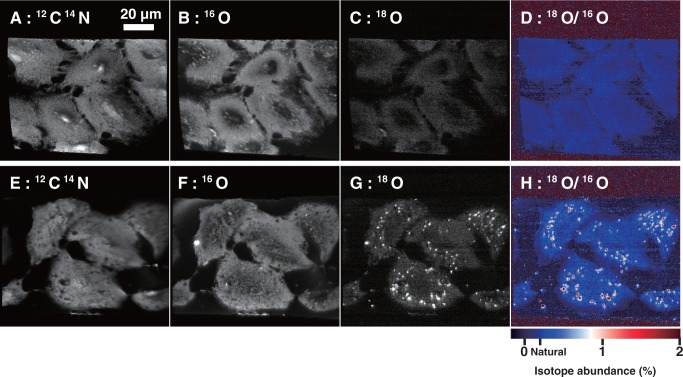
Isotope imaging of A549 cells transfected with unlabeled (**A–D**) or ^18^O-labeled control siRNA (**E–H**). Distribution of (A and E) ^12^C^14^N, (B and F) ^16^O and (C and G) ^18^O. (D and H) ^18^O/^16^O isotopographs. The isotopic abundance of ^18^O is indicated by the color scale.

**Figure 5.**
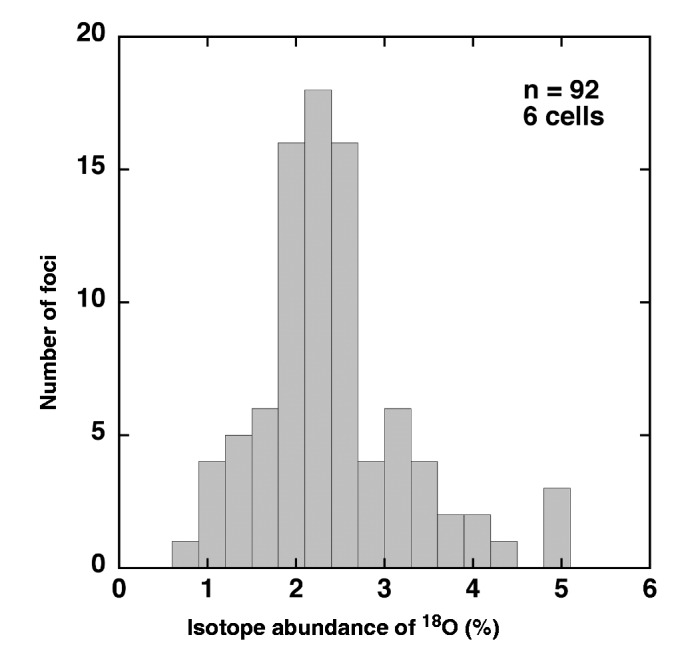
Histogram of the ^18^O isotope abundance of the foci in Figure 4H.

We set out to use isotope microscopy to generate a 2D image of the distribution of ^18^O in A549 cells transfected with ^18^O-labeled RNA (isotopic abundance of ^18^O = 11.77%). The ^12^C^14^N images (Figure 4A and E), which reflect the presence of organic material, clearly show the shapes of the cells. The ^16^O and ^18^O images (Figure 4B, C and F), which reflect the presence of oxygen-containing materials such as proteins and carbohydrates (natural isotopic abundance of ^18^O ≈ 0.2%), also show the shapes of the cells. All of these images (Figure 4A–C, E and F) show a more-or-less uniform distribution of ^12^C^14^N, ^16^O and ^18^O, reflecting the natural abundance of these isotopes. In contrast, the ^18^O image (Figure 4G) and the ^18^O/^16^O ratio image, or isotopograph (Figure 4H), show many bright foci indicating ^18^O enrichment in the cytoplasm around the nucleus, presumably in endosomes. The ^18^O enrichment in the foci was up to 5% above the natural abundance, whereas the ^18^O in the cytoplasm surrounding the foci and in the nucleus remained at its natural abundance (Figure 5). The isotopographs (Figure 4D and H) show the relative abundance of ^18^O in color-coded form, with red indicating the highest abundance. Similar localization patterns of fluorescently labeled siRNA have been reported by Harborth *et al.* (34). These results provide corroborating evidence that ^18^O-labeled RNA was taken up by the cells and accumulated in endosomes, and that the localization of the ^18^O-labeled RNA in the cells was correctly detected by isotope microscopy.

As with the IRMS study, we assume that the observed ^18^O signal was not only from intact RNA but also from degradation products. However, RNA introduced into cells with a transfection agent is reported to be detectable 48 h after transfection (35). Because we used the transfection agent Lipofectamine, it is likely that the ^18^O signals detected with SIMS represent at least some intact RNA. As far as we know, this is the first application of isotope microscopy to the imaging of biological samples containing ^18^O-labeled compounds. Previous localization studies of RNA have usually been performed with fluorescently labeled RNA. However, fluorescence methods have disadvantages. One is the presence of covalently attached fluorophores, which not only make the labeled molecule chemically different from the unlabeled molecule, but in addition are usually bulky and hydrophobic and thus alter all the more the physicochemical properties of the labeled molecule. Another disadvantage is the possible loss of the fluorophore from the labeled molecule. In contrast, ^18^O-labeled RNA appears to be both chemically and biologically equivalent to the corresponding unlabeled RNA. In addition, any desired subset of phosphate groups in the oligomer, or all of them, can be labeled at will. These represent distinct advantages of ^18^O over fluorescent labels in cellular localization analysis. Imaging technology that combines ^18^O labeling with isotope microscopy is expected to be a useful biological and pharmaceutical research tool.

## CONCLUSIONS

We have prepared RNA labeled with one nonbridging ^18^O atom in each phosphate group by using ^18^O water as the oxygen donor to phosphate during the oxidation step of the synthetic cycle. The ^18^O label was stable under physiological conditions for at least 28 days and the ^18^O-labeled RNA was physicochemically and biologically indistinguishable from the corresponding unlabeled RNA by electrophoresis, CD, *T*_m_ and RNAi activity. These properties of ^18^O-labeled RNA make it suitable for use as a tracer in basic and clinical studies of RNA drugs such as antisense oligomers, siRNA, miRNA and aptamers. ^18^O-labeled RNA lends itself to the quantification of RNA in blood by IRMS and to the imaging of the intracellular distribution of the RNA by isotope microscopy. These methods have the potential to avoid the present difficulties with the quantification and visualization of RNA in biological samples by radioisotope and fluorescent labeling, and we expect them to contribute the development of RNA therapeutics.

## SUPPLEMENTARY DATA

Supplementary Data are available at NAR Online: Supplementary Table 1 and Supplementary Figures 1–4.

Supplementary Data
