# Supplementary material for: Synthesis of 18O-labeled RNA for application to kinetic studies and imaging
Source: Nucleic Acids Res. 2013 Apr 30;41(12):e126. doi: 10.1093/nar/gkt344 (PMC3695515; doi:10.1093/nar/gkt344)
Supplement: Supplementary Data [file supp_41_12_e126__index.html]

Synthesis of 18O-labeled RNA for application to kinetic studies and imaging — Synthesis of 18O-labeled RNA for application to kinetic studies and imaging — Supplementary Data 

# Synthesis of 18O-labeled RNA for application to kinetic studies and imaging

## Supplementary Data

files

**Files in this Data Supplement:**

- Supplementary Data - pdf file
