# Supplementary material for: Synthesis of 18O-labeled RNA for application to kinetic studies and imaging
Source: Nucleic Acids Res. 2013 Apr 30;41(12):e126. doi: 10.1093/nar/gkt344 (PMC3695515; doi:10.1093/nar/gkt344)
Supplement: Supplementary Data [file supp_gkt344_nar-03422-met-g-2012-File009.pdf]

Supplementary Data

Synthesis of ^{18}O-labeled RNA for application to kinetic studies and imaging

Tomohiro Hamasaki[1], Takahiro Matsumoto[1,2], Naoya Sakamoto[3], Akiko Shimahara[1], Shiori Kato[1], Ayumi Yoshitake[1], Ayumi Utsunomiya[1], Hisayoshi Yurimoto[3], Esteban C Gabazza[1,2], and Tadaaki Ohgi[1,*].

[1]BONAC Corporation, BIO Factory 4F, 4-1488 Aikawa, Kurume, Fukuoka 839-0861, Japan; [2]Department of Immunology, Mie University Graduate School of Medicine, 2-174 Edobashi, Tsu, Mie 514-8507, Japan; [3]Department of Natural History of Science, Hokkaido University Graduate School of Science, Kita-ku, Sapporo 060-0810, Japan

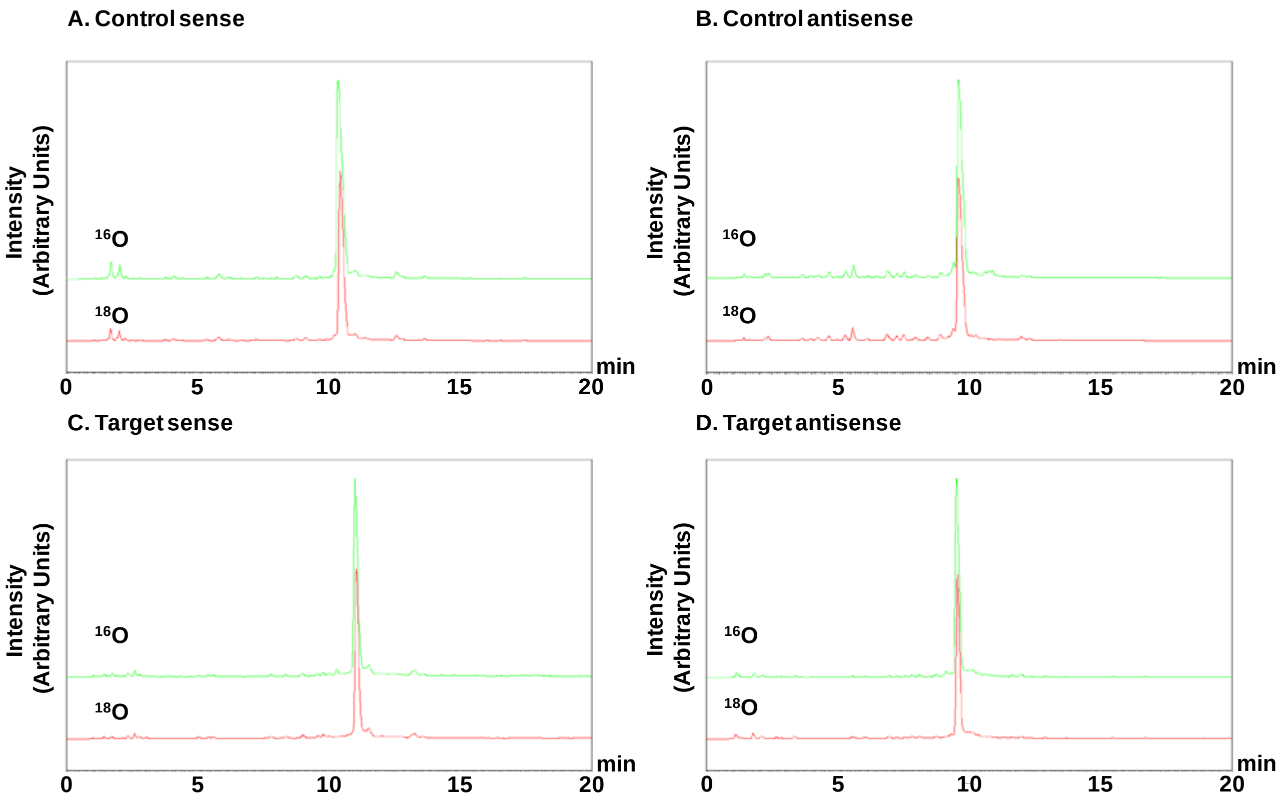

Supplementary Figure S1. HPLC chromatograms of control and target siRNA. Green chromatograms, ^{16}O-RNA; red chromatograms, ^{18}O-labeled RNA.

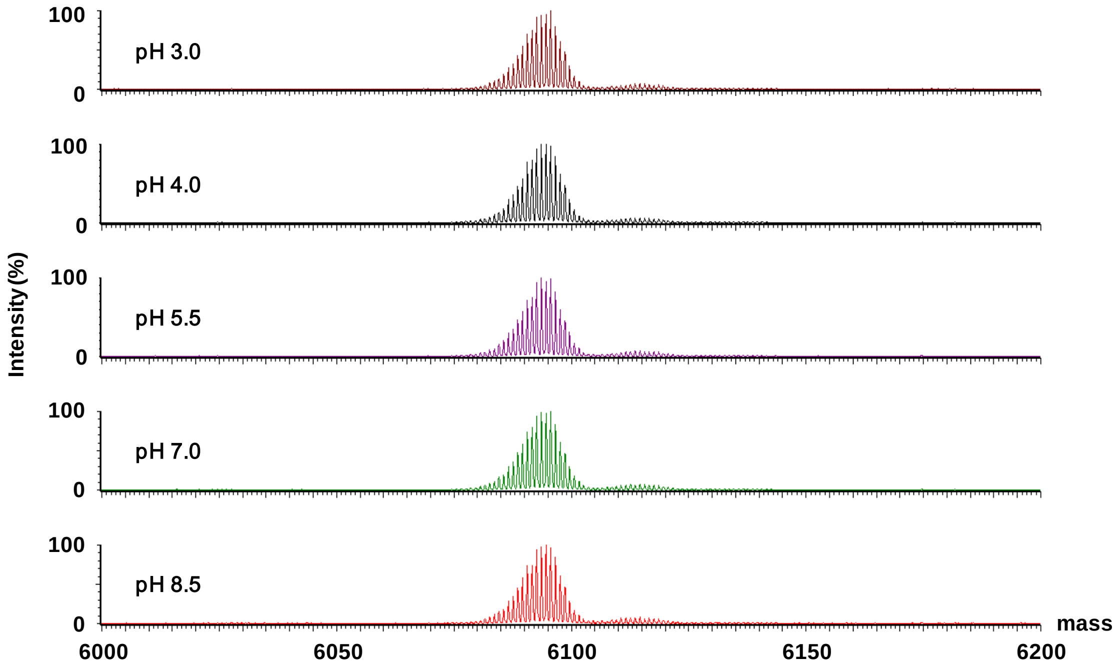

Supplementary Figure S2. LC-ESI-Q-Tof mass spectra of ^{18}O-labeled uridine 20mer RNA after incubation at 37ºC for 28 days at various pH values.

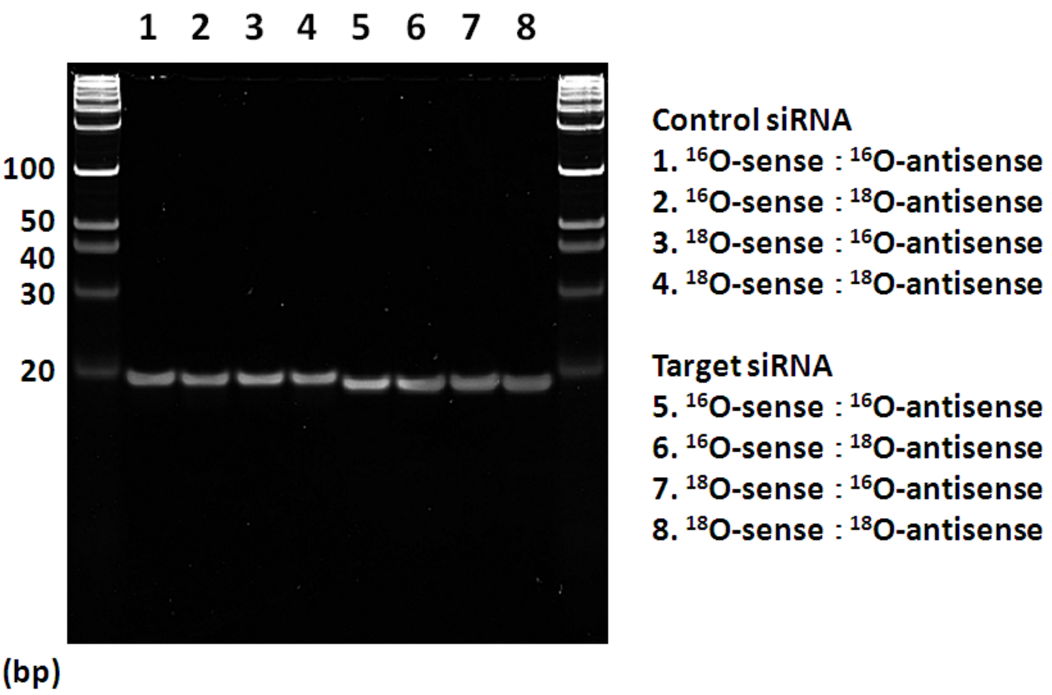

Supplementary Figure S3. Polyacrylamide gel electrophoresis of control and target siRNA. RNA was analyzed on a 15% gel stained with ethidium bromide. siRNA Ladder Marker (Takara Bio) was used as a size marker for double-stranded RNA.

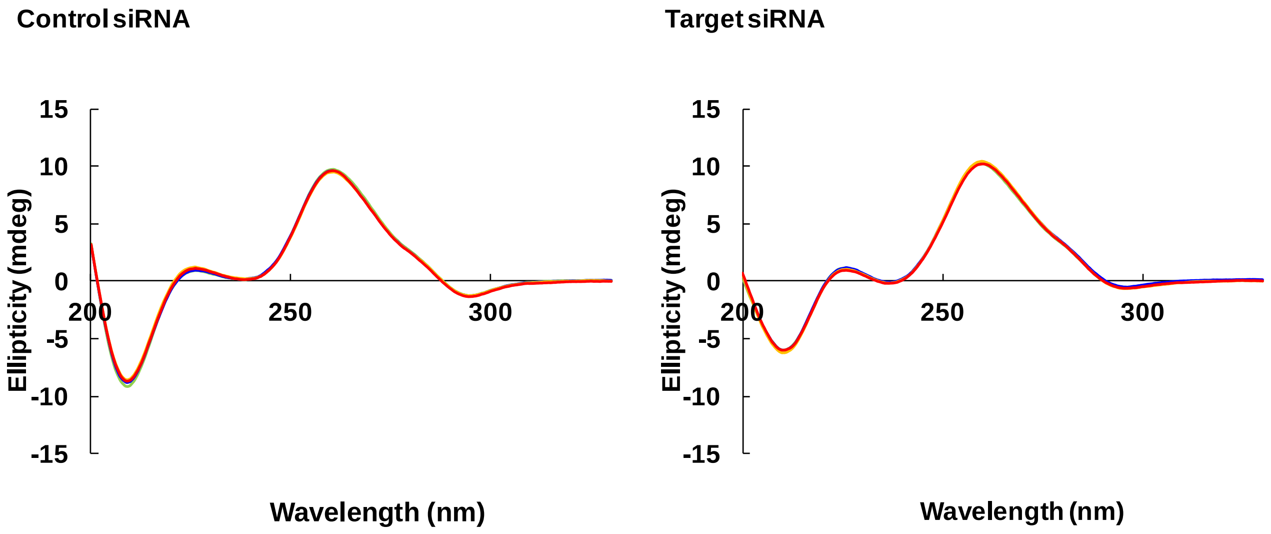

Supplementary Figure S4. CD spectra of 10 μM control (left panel) and target (right panel) siRNA in 50 mM phosphate buffer, pH 7.5, at 25 °C. The sense/antisense strand combinations are shown as follows: green spectra, $^{16}O/^{16}O$-siRNA; blue spectra, $^{16}O/^{18}O$-siRNA; yellow spectra, $^{18}O/^{16}O$-siRNA; red spectra, $^{18}O/^{18}O$-siRNA.

Supplementary Table S1. T_m values (ºC) of control and target siRNA.

	control siRNA	Target siRNA
**^{16}O-sense : ^{16}O-antisense**	**67.69**	**67.16**
**^{16}O-sense : ^{18}O-antisense**	**67.52**	**67.25**
**^{18}O-sense : ^{16}O-antisense**	**67.20**	**66.66**
**^{18}O-sense : ^{18}O-antisense**	**67.10**	**66.66**
